# Phosphatase Activities of a Highly Stable High-Molecular-Mass Multiprotein Complex Isolated from Different Organs of the Sea Cucumber *Paracaudina chilensis*

**DOI:** 10.3390/ijms27146533

**Published:** 2026-07-22

**Authors:** Svetlana E. Soboleva, Nadejda A. Maltseva, Pavel S. Dmitrenok, Georgy A. Nevinsky

**Affiliations:** 1 Institute of Chemical Biology and Fundamental Medicine, Siberian Division of Russian Academy of Sciences, Lavrentiev Ave. 8, Novosibirsk 630090, Russia; 2 G. B. Elyakov Pacific Institute of Bioorganic Chemistry, Far Eastern Branch of the Russian Academy of Sciences, 159 Pr. 100 let Vladivostoku, Vladivostok 690022, Russia

**Keywords:** model of body regeneration, sea cucumber *Paracaudina chilensis*, stable protein complexes, phosphatase activity of the complexes

## Abstract

In recent years, a novel class of highly stable multiprotein complexes, with molecular masses ranging from 1 to 2 MDa, has been identified in human milk, placenta, sea urchin eggs, and sea cucumbers. These complexes exhibit extraordinary stability, dissociating only under stringent conditions involving 8 M urea, 3 M MgCl_2_, EDTA, and DTT. Previous investigations have demonstrated that complexes derived from different organs of the sea cucumber *Paracaudina chilensis* differ in size, molecular mass, and protein/peptide composition; however, their enzymatic activities have remained unexplored. In the present work, we performed the first systematic analysis of phosphatase activity associated with highly stable complexes isolated from five organs of *P. chilensis*: the body wall, gonads, respiratory trees, intestine, and coelomic fluid. Complexes were purified via gel filtration chromatography on Sepharose 4B, followed by ultracentrifugation. Phosphatase activity was determined spectrophotometrically by monitoring the hydrolysis of *p*-nitrophenyl phosphate. Our results indicate that all five complexes harbor phosphatases with optimal pH values spanning 7.0 to 10.0. Alkaline phosphatases (pH 9.0–10.0) displayed pronounced organ specificity: maximal activity was observed in the intestinal complex, whereas minimal activity was detected in the gonadal complex. Phosphatase activity in complexes from the body wall and respiratory trees exhibited a bell-shaped dependence on Mg^2+^ concentration, with optima at 5 mM and 1 mM, respectively; in contrast, activity in the intestinal and coelomic fluid complexes increased to a plateau at 5–10 mM Mg^2+^. Ca^2+^ ions predominantly inhibited activity, with the notable exception of the intestinal complex, where they exerted no effect on hydrolysis. EDTA treatment resulted in complete inactivation of the enzymes in most complexes; however, intestinal activity was retained at 50% even at high chelator concentrations, suggesting the presence of a metal-independent phosphatase. Collectively, these data indicate that the stable multiprotein complexes of *P. chilensis* contain an organ-specific repertoire of phosphatases that differ in pH optimum, metal ion dependence, and inhibitor sensitivity. These findings open new avenues for understanding the roles of such complexes in organ-specific physiological functions and regenerative mechanisms in echinoderms.

## 1. Introduction

It is known that many important biological processes can be mediated by the action of various multiprotein complexes [1]. Numerous biological processes are governed by the functioning of diverse specific enzymes, proteins, peptides, and other compounds within cells and biological fluids, which often form stable or transient multifunctional complexes. The operation of such specific complexes ensures a significant increase in the efficiency, specificity, and rate of metabolic pathways [2]. The association of various proteins, enzymes, DNA, RNA, and other molecules can lead to the formation of very important polyfunctional complexes that exhibit expanded biological functions compared with their individual components. Several stable protein complexes with diverse functions have been described [3,4,5,6,7,8,9,10,11]. Ribosomes are well-known, highly stable complexes that comprise multisubunit ribonucleoprotein assemblies responsible for protein synthesis [12]. Moreover, ribosomes can form a variety of additional modified protein complexes with other important functions [13,14,15,16,17,18]. Complexes of associated specific proteins bound to human placental membranes have been investigated using SDS-PAGE and MALDI mass spectrometry [19]. In total, 733 different specific proteins were found to be incorporated into thirty-four protein complexes. Therefore, the study of stable, yet undescribed polyfunctional complexes is of particular interest.

A novel type of highly stable multiprotein complex has recently been identified in human breast milk [20], human placenta [21,22], sea urchin eggs [23], and the sea cucumbers *Eupentacta fraudatrix* [24] and *Paracaudina chilensis* [25]. Stable protein complexes derived from human breast milk and sea urchin eggs have molecular weights (MWs) of 1000 ± 100 kDa [20,21,22,23]. In contrast, the highly stable protein complexes from the sea cucumbers *E. fraudatrix* [24] and *P. chilensis* [25] possess MWs of approximately 1500–2000 kDa. All these complexes contain a diverse array of proteins with MWs above 10 kDa, as well as peptides with MWs below 10 kDa [20,21,22,23,24,25]. In the case of the sea cucumber *P. chilensis*, a comparative analysis of highly stable complexes isolated from various organs, including coelomic fluid, body wall, respiratory trees, gut, and gonads, was performed for the first time [25]. The results showed that each organ contains its own specific highly stable complex, which can be distinguished from complexes of other organs by size (as determined by electron microscopy), MW (in the range of 1.2–2.2 MDa according to gel filtration data), and the relative content and types of proteins (>10 kDa) and peptides (<10 kDa) (as determined by MALDI mass spectrometry). Depending on the organ, the number of distinct specific peptides present in these complexes from five different organs ranged from 55 to 104. All these recently identified unusual protein complexes exhibit high stability and can be effectively dissociated only in the presence of 8.0 M urea containing 0.1 M EDTA, 1.0–3.0 M MgCl_2_, and DTT [20,21,22,23,24,25]. The formation of such complexes cannot be random. It has been shown that the various components of these complexes form electrostatic and hydrogen bonds, as well as metal-dependent contacts and disulfide (S–S) linkages [20,21,22,23,24,25].

These complexes contain various enzymes on their surfaces. Only the activities that have been investigated in each complex are described below. In the milk complex, Mg^2+^-dependent DNase and metal-independent amylase were detected [20], whereas the sea urchin egg complex was found to exhibit phosphatase activity [23]. The complex from the whole organism of the sea cucumber *E. fraudatrix* was examined for two activities: DNase and protease [24]. In the case of the placental complex, additional possible enzymatic activities were analyzed [21,22]. This complex demonstrated nine different catalytic activities: amylase, phosphatase, ATPase, DNase, RNase, protease, catalase, H_2_O_2_-independent oxidoreductase, and H_2_O_2_-dependent peroxidase.

However, it is possible that such unusual, large, and highly stable complexes may possess a considerably greater number of different enzymatic activities. With this in mind, it was of interest to determine whether complexes from different organs of *P. chilensis* differ not only in protein and peptide composition, but also in the catalytic activity of their enzymes.

Phosphatases are critically important enzymes in living organisms. The best-known phosphatases, including alkaline phosphatases, remove phosphate groups (dephosphorylation) from many types of molecules in the body, including proteins, nucleotides, polysaccharides, and alkaloids [26]. Phosphatases have been classified on the basis of substrate specificity and amino acid sequence homology of their catalytic domains [27,28]. All these different phosphatases have been divided into 104 distinct enzyme families, including acid phosphatases, alkaline phosphatases, the endonuclease/exonuclease/phosphatase family, phosphotransferases, and protein phosphatases. Despite classification into more than one hundred families, all phosphatases catalyze the same general hydrolysis reaction [27,28]. Alkaline phosphatase is a homodimeric enzyme with a molecular mass of 86 kDa. Each monomer contains five cysteine residues, two Zn^2+^ atoms, and one Mg^2+^ atom, all of which are essential for its catalytic function [26]. Alkaline phosphatases are most active at alkaline pH values [29]. The enzyme is found in various eukaryotic and prokaryotic organisms, performing similar functions but existing in different structural forms. Alkaline phosphatase is also present in the periplasmic space of *E. coli*. In mammals, it exists in several forms depending on the tissue of origin. Alkaline phosphatase plays an important role in liver metabolism and skeletal development. The relative concentration of phosphatase in the bloodstream is used by clinicians as a biomarker for hepatitis or osteomalacia [30]. Alkaline phosphatase influences inflammatory responses in patients with chronic kidney disease and is directly linked to anemia resistant to erythropoiesis-stimulating agents [31]. Intestinal alkaline phosphatases regulate pH and ATP hydrolysis in the rat duodenum [32]. Acid phosphatases are subdivided into two subclasses–metal-dependent and metal-independent. Metal-independent acid phosphatases are found in various species such as fungi, bacteria, parasites, and plants, and most of them share structural similarity with mammalian acid phosphatases [33,34].

In addition to magnesium-dependent phosphatases, calcium-dependent phosphatases have been identified in various organisms [33,34,35,36,37,38,39,40,41,42,43]. Various calcium-dependent phosphatases are critical enzymes in diverse biochemical pathways in different cell types. These phosphatases play a central role in controlling signal transduction. It is well known that phosphatases exhibit specificity for removing phosphate groups from tyrosine or serine/threonine residues [33,34,35,36,37,38,39,40,41,42,43].

Given the importance of various phosphatases for numerous processes in living organisms, in the present work we performed, for the first time, an analysis of the phosphatase activity of several stable complexes from different organs of the sea cucumber *P. chilensis*. It was shown that the complexes from different organs differ in their content of various phosphatases.

## 2. Results

### 2.1. Preparation of Different Organs Homogenates and Protein Complexes

Samples of the whole organisms of the sea cucumber *P. chilensis* and different organs (respiratory trees, gut, upper body wall, gonads, and coelomic fluid) were subjected to careful homogenization and used for the isolation of several stable protein complexes [25]. Homogenates of various organs were subjected to FPLC gel filtration using Sepharose 4B, which effectively separates molecules with molecular weights (MWs) ranging from 60 to 20,000 kDa. Representative gel filtration profiles are given in [25] and shown in Supplementary Figure S1. The protein complexes exhibited different MWs ranging from 1.2 to 1.8 megaDaltons (MDa): whole organism, 1.8 ± 0.1; body wall, 1.7 ± 0.2; gonads, 1.7 ± 0.2; gut, 1.5 ± 0.1; respiratory trees, 1.4 ± 0.1 and 1.2 ± 0.2; coelomic fluid [25].

Following gel filtration, each complex was subjected to additional purification by ultracentrifugation to eliminate co-eluting vesicles. Supplementary Figure S2 demonstrates the samples obtained from the entire holothurian body and visceral organs, showing protein complexes of different sizes using transmission electron microscopy (a description of the complex isolation procedure and analysis by transmission electron microscopy is provided in Supplementary Methods S1).

All detailed data concerning the isolation and characterization of the obtained protein complexes and their components using SDS-PAGE, MALDI mass spectrometry, and other methods are described in [25]. The SDS-PAGE analysis of all protein complexes is shown in Figure S3. In addition to SDS-PAGE, the molecular weights of proteins ranging from 10 to 20 kDa were determined by MALDI mass spectrometry (Supplementary Table S1). The molecular masses of peptides (<10 kDa) within stable complexes isolated from various tissues were assessed by MALDI mass spectrometry (Supplementary Table S2). The final stable multiprotein complexes were used to analyze their phosphatase activity.

### 2.2. Phosphatase Activities of Complexes from Different Organs of Sea Cucumber

**Figure 1:**
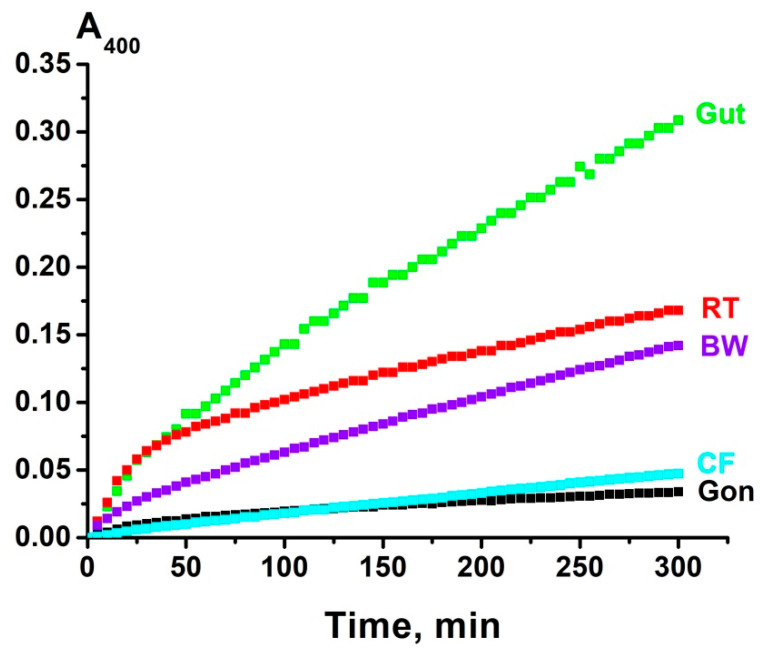
Representative examples of phosphatase activity determination based on the increase in A_400_ resulting from the hydrolysis of *p*-nitrophenyl phosphate in the presence of individual complexes (0.06 mg/mL) at different pH values. All designations are provided on the panel: complexes corresponding to coelomic fluid (CF; pH = 5.5, 6.0, and 7.0), body wall (BW; pH = 5.0), and respiratory trees (RT; pH = 9.5). The error in initial rate determination from two independent experiments did not exceed 10%.

Phosphatase activity of the complexes was estimated by monitoring the increase in absorbance at 400 nm (A_400_) resulting from the hydrolysis of *p*-nitrophenyl phosphate. All complexes hydrolyzed *p*-nitrophenyl phosphate at pH 7.5 and at other pH values, albeit at different rates. Several typical kinetic curves showing relative phosphatase activity in the presence of five individual preparations of the complexes isolated from different organs are presented in Figure 1. Such kinetic curves at different pH levels were obtained for each complex.

### 2.3. Optimal pH of Substrate Hydrolysis

**Figure 2:**
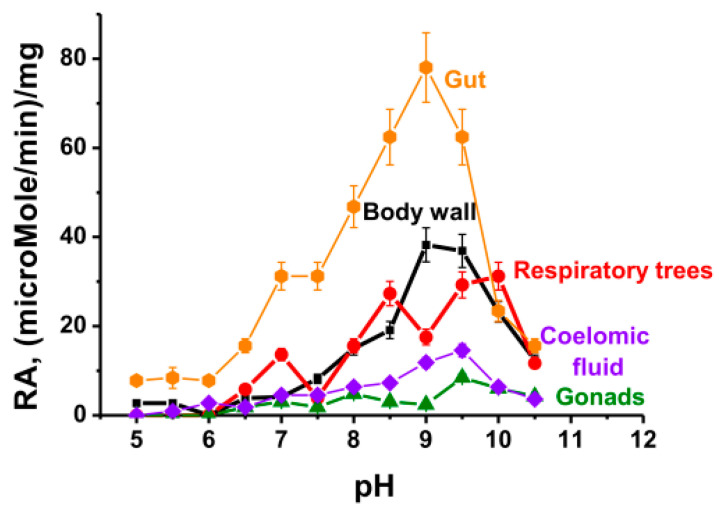
The data on the relative phosphatase activity of the complexes from different organs at various pH values in the absence of metal ions. According to two independent experiments, the error in determining the relative activity did not exceed 8–10%.

Canonical phosphatases can have many different optimal pH values [26,27,28,29,30,31,32,33]. Therefore, it was important to evaluate the optimal pH values of phosphatases corresponding to complexes from different organs of the sea cucumber. Initially, an analysis was made of the optimal pH values in the absence of metal ions (Figure 2).

Analyzing the data on the relative phosphatase activity of complexes from different holothurian organs at various pH values in the absence of external metal ions, it should be noted that these complexes contain internal metal ions [44], which can potentially activate the phosphatases in the complexes.

Notably, all five complexes contain phosphatases with optimal pH values ranging from 9 to 9.5. The activity of these alkaline phosphatases decreases depending on the organ in the following order: gut > body wall ≥ respiratory trees > coelomic fluid > gonads (Figure 2). Furthermore, the complexes from the gut and respiratory trees exhibit phosphatase activity at approximately pH 7.0. Moreover, in the case of respiratory trees, well-defined activity is observed at pH 8.5 and from 9.5 to 10. Additionally, while the complex from the gut shows maximal activity at pH 9.0, the complexes from coelomic fluid and gonads exhibit their maximum at pH 9.5. These data indicate that during complex formation in different organs of the sea cucumber, distinct phosphatases are incorporated into the complexes.

### 2.4. Dependence of Phosphatase Activity on Metal Ions

Phosphatases from different organisms may differ in their dependence on Mg^2+^ ions: some require a specific concentration for maximal activity, while others are metal-independent [35,36,37,38,39,40,41,42,43]. Therefore, it was of interest to evaluate dependence of phosphatase activity of complexes from different organs on metal ion concentrations. The dependence of phosphatase activity on Mg^2+^, Ca^2+^, and EDTA for complexes isolated from various organs was assessed at pH values corresponding to the maximum activities of the complexes under analysis.

**Figure 3:**
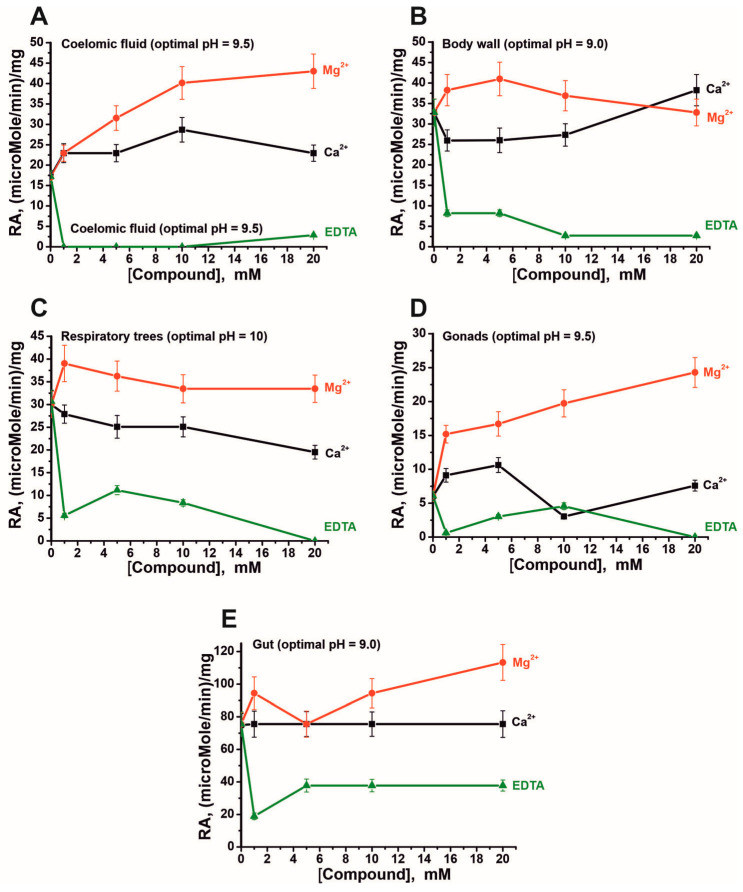
Generalized data on the relative phosphatase activity of the complexes from different organs in the presence of MgCl_2_, CaCl_2_ and EDTA at different concentrations: coelomic fluid (**A**), body wall (**B**), respiratory trees (**C**), gonads (**D**), and gut (**E**). According to two independent experiments, the error in determining the relative phosphatase activity did not exceed 10–20%.

As shown in Figure 3, at optimal pH values, all phosphatases within the five complexes are magnesium-dependent. It is known that, in some cases, metal ion dependencies exhibit a bell-shaped pattern. However, this type of dependence on magnesium ion concentration was observed only for the body wall and respiratory tree complexes. Notably, the plateau or maximum activity attainment for complexes from different organs differs markedly: 1 mM (respiratory tree), 5 mM (body wall and gut), and 10 mM (coelomic fluid). In the case of gonads, a sharp increase in activity is observed at 1 mM, followed by a further gradual increase in activity.

Calcium ions either have no effect on phosphatase activity for the gut complex (Figure 3E) or weakly activate the phosphatase activity of the complex from the gonad at low concentrations (Figure 3D). For the remaining complexes, inhibition of phosphatase activity by Ca^2+^ ions is predominantly observed.

Particular attention should be paid to the effect of EDTA on the phosphatase activity of the complexes. In most cases, almost complete inhibition of phosphatase activity is observed at very high EDTA concentrations. However, for some complexes (respiratory tree and gonads), an increase in phosphatase activity is observed at certain EDTA concentrations. Nevertheless, only in the case of the gut complex does EDTA inhibit the activity by only about 50%, even at very high concentrations. This may indicate that metal-independent phosphatases are present in the complexes from these organs.

## 3. Discussion

Sea urchin eggs, human placenta, and human milk, as noted above, contain very stable protein complexes with MWs of 1000 ± 100 kDa [20,21,22,23], while in the sea cucumber *E. fraudatrix*, a multiprotein complex having MWs of 1500–2000 kDa was found [24]. The existence of extremely stable multiprotein complexes testifies that their formation cannot be the result of random association of various proteins [20,21,22,23]. The above studies investigated stable complexes without isolating and comparing complexes from different organs [20,21,22,23] or from the entire sea cucumber *E. fraudatrix* organism [24]. At the same time, it was of interest to know whether very stable multiprotein complexes in different organs of the same organism may be different and, if they are, how they differ in their protein, peptide, and enzyme composition and various properties in comparison with all individual components and enzymes of different organs.

Recently, we conducted the first analysis of highly stable multiprotein complexes from different organs (upper body wall, gonads, respiratory trees, gut, and coelomic fluid) of the sea cucumber *P. chilensis* and obtained unexpected results [25]. It turned out that according to gel filtration and electron microscopy data, all stable complexes from different organs of *P. chilensis* have different sizes, MWs, and compositions. Each of the complexes consists of many different proteins with molecular weights >10 kDa. In addition to large proteins, all protein complexes contain different numbers (from 55 to 104) of various <10 kDa oligopeptides [25].

These findings emphasize that very different stable protein-enzyme complexes may be associated with the performance of specific functions by each organ and can be formed in different organs in a specific manner using different peptides, proteins and enzymes. It is known that different organs usually contain not only the same enzymes but also some organ-specific ones. Taking this into account, in this work we analyzed for the first time the phosphatases activity of stable complexes from different organs.

Several phosphatases have been detected in the complexes from each organ that catalyze the reaction in the absence of any metal ions (Figure 2). However, as shown earlier, the stable complexes of sea cucumbers contain magnesium, calcium, and certain other metal ions bound to the complex components [44]. Given this, it remains unclear whether the phosphatases active in the absence of exogenous metal ions are metal-dependent or metal-independent. When analyzing the dependence of phosphatase activity on Ca^2+^ ions, one should take into account the literature data. For example, some phosphatases are Mg^2+^-dependent enzymes, but Ca^2+^ and other metal ions still activate these enzymes, although to a much lesser extent [38]. Taking this into account, it cannot be ruled out that some of the phosphatases in the protein complexes may be Ca^2+^-dependent enzymes, while others are Mg^2+^-dependent and can also use Ca^2+^ ions as a cofactor.

As shown in Figure 3, alkaline phosphatases exhibiting maximum activity at pH 9.0–10.0 differ among organ with respect to the optimal concentrations of magnesium and calcium ions. Furthermore, the activity of some of these phosphatases is not completely inhibited even at high concentrations of EDTA. This may suggest that, alongside metal-dependent phosphatases, these complexes contain less active metal-independent enzymes. It is unequivocally clear, nevertheless, that the stable complex from the gut contains a metal-independent alkaline phosphatase capable of hydrolyzing the substrate at pH 9.0, since the activity of this complex is inhibited by EDTA at very high concentrations by only about half.

Several phosphatases from echinoderms have been described to date. An alkaline phosphatase with a molecular mass of 166 ± 9 kDa and an optimal pH of 11 was isolated from the intestine of the sea cucumber *Stichopus japonicus* [45]. The enzyme activity was enhanced in the presence of Mg^2+^ and inhibited by Ca^2+^, Zn^2+^, and EDTA at concentrations of 1–10 mM, suggesting its dependence on magnesium ions [46]. An alkaline phosphatase from the eggs of the sea urchin *Strongylocentrotus intermedius* is a homodimer with a molecular mass of 150 kDa and exhibits maximum activity at pH 8.1–8.5 [46]. Its activity was stimulated by Mg^2+^, Ca^2+^, Mn^2+^, and DTT, whereas Zn^2+^, Cu^2+^, Cd^2+^, Pb^2+^, Ni^2+^, and EDTA acted as inhibitors. This enzyme was characterized by high salt tolerance and the ability to hydrolyze substrates in seawater [46]. In addition, an alkaline phosphatase has been identified in the starfish *Astropecten bispinosus* (*Asterias bispinosa*) [47]. Apart from echinoderms, phosphatases have also been isolated from Atlantic cod (*Gadus morhua*) [48], the bivalve mollusk *Ruditapes philippinarum* [49], and deep-sea fish [50]. Overall, the phosphatases characterized in the present work show similarities to the previously described echinoderm enzymes.

Thus, the data presented allow us to conclude that the non-random formation of stable complexes occurs in such a way that complexes from different organs can incorporate distinct phosphatases with different optimal pH values and metal ion concentrations. Consequently, these highly multifunctional complexes are of particular interest for future research and for understanding their expanded biological functions in each organ.

## 4. Materials and Methods

### 4.1. Reagents and Samples

High-purity reagents (Tris, SDS, various salts, EDTA, DTT, glycerol, NH_4_HCO_3_, urea, and some other compounds) were obtained from Sigma (St. Louis, MO, USA). Sepharose 4B sorbent was from GE Healthcare Life Sciences (New York, NY, USA). Sea cucumbers (holothurians) *Paracaudina chilensis* were collected from Peter the Great Bay in the Sea of Japan. Samples of sea cucumbers were frozen to −40 °C and stored until homogenate preparation. A description of the conditions for finding sea cucumbers in Peter the Great Bay in the Sea of Japan is given in [25].

This study used holothurians, a marine organism, and ethical approval is not currently required for work with fish and other marine and oceanic organisms, including sea cucumbers. Nevertheless, the study was conducted in accordance with the recommendations of the Institutional Ethics Committee of the G.B. Elyakov Pacific Institute of Bioorganic Chemistry, Far Eastern Branch of the Russian Academy of Sciences (Protocol No. 02/24, dated 15 April 2024). The Institute of Chemical Biology and Fundamental Medicine of the Siberian Branch of the Russian Academy of Sciences provided institutional support for our work with marine organisms.

### 4.2. Isolation of Stable Complexes by Gel Filtration

Preparation of organ homogenates from sea cucumbers was performed as described in [25]. Homogenates of respiratory trees, coelomic fluid, body wall, intestine, and gonads were centrifuged at 1.6 × 10^3^× *g* for 55 min, and the supernatants were dialyzed against Milli-Q purified water (Merck KGaA, Darmstadt, Germany). To remove contaminating vesicles and cellular membrane fragments, the supernatants were subjected to ultracentrifugation at 100,000× *g* for 2 h using an Optima XE-90 ultracentrifuge (Beckman Coulter, CA, USA), after which the supernatants were concentrated [25]. The clarified homogenates were further centrifuged at 1.3 × 10^3^× *g* for 15 min at 4 °C and then fractionated by FPLC gel filtration on Sepharose 4B to obtain highly stable protein complexes (1.2–2.2 MDa) [25] (Supplementary Figure S1). To remove any residual vesicular contaminants from the stable multiprotein complex preparations, a second ultracentrifugation step was performed at 100,000× *g* for 1.5 h. The final preparations were used for phosphatase activity assays.

### 4.3. Assay of Phosphatase Activity

The phosphatase activity of stable complexes was analyzed according to [51]. The reaction mixture (100 µL) contained 50 mM Tris-HCl (pH 9.0) (or buffer with a different pH, see below), 0.2 mM *p*-nitrophenyl phosphate, and one of the complexes at a concentration of 0.007–0.06 mg/mL. In some experiments, the reaction mixture additionally contained MgCl_2_, CaCl_2_, or EDTA at concentrations ranging from 0 to 20 mM. During incubation, the optical density (A_400_) of the reaction mixture was measured. All measurements were performed within the linear ranges of the time courses and the complex concentration curves. All pH dependencies of phosphatase activity were analyzed using several different buffers (50 mM): citric acid-NaOH (pH 4.0–5.0), MES-NaOH (pH 5.4–6.6), Tris-HCl (pH 6.0–8.8), and glycine-NaOH (pH 9.0–10.5). To find *k*_cat_ values, the molar extinction coefficient of *p*-nitrophenol was used: ε = 18,300 M^−1^·cm^−1^ [51].

### 4.4. Statistical Analysis

The data are given as mean ± S.E. from at least three experiments for each analysis type.

OriginPro 2019 software (https://www.originlab.com/2019, accessed on 6 November 2025, OriginLab Corporation, Northampton, MA, USA) was used for statistical analysis.

## 5. Conclusions

This article is the first to analyze the phosphatase activity of very stable protein complexes from different organs of the sea cucumber *Paracaudina chilensis* (gut, respiratory trees, body wall, gonads, and coelomic fluid). It was shown that protein complexes from various organs with molecular weights ranging from 1.2 to 2.2 MDa contain several different phosphatases having different optimal pH values (from 7.0 to 10.0) and optimal concentrations of MgCl_2_ and CaCl_2_. Some complexes contain metal-independent phosphatases. Each of the complexes consists of a specific set of phosphatases. It is evident that the nonrandom formation of stable complexes occurs in such a way that different phosphatases with varying optimal pH values and optimal metal ion concentrations are incorporated into the composition of complexes from different organs. Therefore, such highly multifunctional complexes are of particular interest for future research and for understanding their extended biological functions in each organ.

## Data Availability

The data that supports the findings of this study are available within the article and in Supplementary Figure S1 (obtaining of the complex using gel filtration on the Sepharose 4B) and Supplementary Figure S2—transmission electron microscope data of different stable complexes.

## Supplementary Materials

The following supporting information can be downloaded at: https://www.mdpi.com/article/10.3390/ijms27146533/s1.

## Abbreviations

DTT, dithiothreitol; EDTA, ethylenediaminetetraacetic acid; kDa, kilo Daltons; MDa mega Daltons; MW, molecular weight; SDS, sodium dodecyl sulfate; SDS-PAGE, sodium dodecyl sulfate-polyacrylamide gel electrophoresis; MALDI-TOF mass spectrometry-matrix-assisted laser desorption ionization mass spectrometry.

## References

[1] Alberts B. (1998). The cell as a collection of protein machines: Preparing the next generation of molecular biologists. Cell.

[2] Eubel H., Braun H.P., Millar A.H. (2005). Blue-native PAGE in plants: A tool in analysis of protein-protein interactions. Plant Methods.

[3] Aroya S.B., Kupiec M. (2005). The Elg1 replication factor C-like complex: A novel guardian of genome stability. DNA Repair.

[4] Martinez E. (2002). Multi-protein complexes in eukaryotic gene transcription. Plant Mol. Biol..

[5] He L., Yang J., Hao Y., Yang X., Shi X., Zhang D., Zhao D., Yan W., Bie X., Chen L. (2023). DDX20: A Multifunctional Complex Protein. Molecules.

[6] Kim J.J., Kingston R.E. (2022). Context-specific Polycomb mechanisms in development. Nat. Rev. Genet..

[7] Arif A., Yao P., Terenzi F., Jia J., Ray P.S., Fox P.L. (2018). The GAIT translational control system. Wiley Interdiscip. Rev. RNA.

[8] Schulte U., den Brave F., Haupt A., Gupta A., Song J., Müller C.S., Engelke J., Mishra S., Mårtensson C., Ellenrieder L. (2023). Mitochondrial complexome reveals quality-control pathways of protein import. Nature.

[9] Swapna L.S., Mahajan S., de Brevern A.G., Srinivasan N. (2012). Comparison of tertiary structures of proteins in protein-protein complexes with unbound forms suggests prevalence of allostery in signalling proteins. BMC Struct. Biol..

[10] Kohrman D.C., Imperiale M.J. (1992). Simian virus 40 large T antigen stably complexes with a 185-kilodalton host protein. J. Virol..

[11] Valach M., Léveillé-Kunst A., Gray M.W., Burger G.J. (2018). Respiratory chain Complex I of unparalleled divergence in diplonemids. J. Biol. Chem..

[12] Salim D., Gerton J.L. (2019). Ribosomal DNA instability and genome adaptability. Chromosome Res..

[13] Leal G., Comprido D., Duarte C.B. (2014). BDNF-induced local protein synthesis and synaptic plasticity. Neuropharmacology.

[14] Jiao L., Liu Y., Yu X.Y., Pan X., Zhang Y., Tu J., Song Y.H., Li Y. (2023). Ribosome biogenesis in disease: New players and therapeutic targets. Signal Transduct. Target. Ther..

[15] Cech T.R., Steitz J.A. (2014). The noncoding RNA revolution-trashing old rules to forge new ones. Cell.

[16] Gammons M., Bienz M. (2018). Multiprotein complexes governing Wnt signal transduction. Curr. Opin. Cell Biol..

[17] Gammons M.V., Rutherford T.J., Steinhart Z., Angers S., Bienz M.J. (2016). Essential role of the Dishevelled DEP domain in a Wnt-dependent human-cell-based complementation assay. Cell Sci..

[18] Gammons M.V., Renko M., Johnson C.M., Rutherford T.J., Bienz M. (2016). Wnt Signalosome Assembly by DEP Domain Swapping of Dishevelled. Mol. Cell.

[19] Wang F., Wang L., Xu Z., Liang G. (2013). Identification and analysis of multi-protein complexes in placenta. PLoS ONE.

[20] Soboleva S.E., Dmitrenok P.S., Verkhovod T.D., Buneva V.N., Sedykh S.E., Nevinsky G.A. (2015). Very stable high molecular mass multiprotein complex with DNase and amylase activities in human milk. J. Mol. Recognit..

[21] Burkova E.E., Dmitrenok P.S., Sedykh S.E., Buneva V.N., Soboleva S.E., Nevinsky G.A. (2014). Extremely stable soluble high molecular mass multi-protein complex with DNase activity in human placental tissue. PLoS ONE.

[22] Burkova E.E., Dmitrenok P.S., Bulgakov D.V., Ermakov E.A., Buneva V.N., Soboleva S.E., Nevinsky G.A. (2018). Identification of major proteins of a very stable high molecular mass multi-protein complex of human placental tissue possessing nine different catalytic activities. Biochem. Anal. Biochem..

[23] Soboleva S.E., Burkova E.E., Dmitrenok P.S., Bulgakov D.V., Menzorova N.I., Buneva V.N., Nevinsky G.A. (2018). Extremely stable high molecular mass soluble multiprotein complex from eggs of sea urchin *Strongylocentrotus intermedius* with phosphatase activity. J. Mol. Recognit..

[24] Timofeeva A.M., Kostrikina I.A., Dmitrenok P.S., Soboleva S.E., Nevinsky G.A. (2021). Very Stable Two Mega Dalton High-Molecular-Mass Multiprotein Complex from Sea Cucumber *Eupentacta fraudatrix*. Molecules.

[25] Soboleva S.E., Poletaeva J.E., Dmitrenok P.S., Bulgakov D.V., Ryabchikova E.I., Nevinsky G.A. (2025). Very Stable High-Molecular-Mass Multiprotein Complexes in Different Organs of the Sea Cucumber *Paracaudina chilensis*. Molecules.

[26] Millán J.L. (2006). Alkaline Phosphatases: Structure, substrate specificity and functional relatedness to other members of a large superfamily of enzymes. Purinergic Signal..

[27] Sacco F., Perfetto L., Castagnoli L., Cesareni G. (2012). The human phosphatase interactome: An intricate family portrait. FEBS Lett..

[28] Alkaline Phosphatase (EC 3.1.3.1)|Protein Target—PubChem. https://pubchem.ncbi.nlm.nih.gov/protein/EC:3.1.3.1.

[29] Tamás L., Huttová J., Mistrk I., Kogan G. (2002). Effect of carboxymethyl chitin-glucan on the activity of some hydrolytic enzymes in maize plants. Chem. Pap..

[30] Alkaline Phosphatase Level Test (ALP). Healthline.

[31] Badve S.V., Zhang L., Coombes J.S., Pascoe E.M., Cass A., Clarke P., Ferrari P., McDonald S.P., Morrish A.T., Pedagogos E. (2015). Association between serum alkaline phosphatase and primary resistance to erythropoiesis stimulating agents in chronic kidney disease: A secondary analysis of the HERO trial. Can. J. Kidney Health Dis..

[32] Ham M., Mizumori M., Watanabe C., Wang J.H., Inoue T., Nakano T., Guth P.H., Engel E., Kaunitz J.D., Akiba Y. (2010). Endogenous luminal surface adenosine signaling regulates duodenal bicarbonate secretion in rats. J. Pharmacol. Exp. Ther..

[33] Araujo C.L., Vihko P.T. (2013). Structure of Acid phosphatases. Methods Mol. Biol..

[34] Zhao L., Wang L., Chi C., Lan W., Su Y. (2017). The emerging roles of phosphatases in Hedgehog pathway. Cell Commun. Signal..

[35] D’Alcantara P., Schiffmann S.N., Swillens S. (2003). Bidirectional synaptic plasticity as a consequence of interdependent Ca^2+^-controlled phosphorylation and dephosphorylation pathways. Eur. J. Neurosci..

[36] Woolfrey K.M., Dell’Acqua M.L. (2015). Coordination of Protein Phosphorylation and Dephosphorylation in Synaptic Plasticity. J. Biol. Chem..

[37] Hogan P.G., Li H. (2005). Calcineurin. Curr. Biol..

[38] Hemenway C.S., Heitman J. (1999). Calcineurin. Structure, function, and inhibition. Cell Biochem. Biophys..

[39] Hinke S.A., Navedo M.F., Ulman A., Whiting J.L., Nygren P.J., Tian G., Jimenez-Caliani A.J., Langeberg L.K., Cirulli V., Tengholm A. (2012). Anchored phosphatases modulate glucose homeostasis. EMBO J..

[40] Dell’Acqua M.L., Smith K.E., Gorski J.A., Horne E.A., Gibson E.S., Gomez L.L. (2006). Regulation of neuronal PKA signaling through AKAP targeting dynamics. Eur. J. Cell Biol..

[41] Lim Z.W., Chen W.L. (2020). Exploring the association of Bone Alkaline Phosphatases and Hearing Loss. Sci. Rep..

[42] Piol D., Tosatto L., Zuccaro E., Anderson E.N., Falconieri A., Polanco M.J., Marchioretti C., Lia F., White J., Bregolin E. (2023). Antagonistic effect of cyclin-dependent kinases and a calcium-dependent phosphatase on polyglutamine-expanded androgen receptor toxic gain of function. Sci. Adv..

[43] Bastan R., Eskandari N., Sabzghabaee A.M., Manian M. (2014). Serine/Threonine phosphatases: Classification, roles and pharmacological regulation. Int. J. Immunopathol. Pharmacol..

[44] Zaksas N.P., Timofeeva A.M., Dmitrenok P.S., Soboleva S.E., Nevinsky G. (2022). Comparison of the Content of Several Elements in Seawater, Sea Cucumber *Eupentacta fraudatrix* and Its High-Molecular-Mass Multiprotein Complex. Molecules.

[45] Wu H.-T., Li D.-M., Zhu B.-W., Cheng J.-H., Sun J.-J., Wang F.-L., Yang Y., Song Y.-K., Yu C.-X. (2013). Purification and characterization of alkaline phosphatase from the gut of sea cucumber *Stichopus japonicus*. Fish. Sci..

[46] Seitkalieva A.V., Menzorova N.I., Vakorina T.I., Dmitrenok P.S., Rasskazov V.A. (2017). A new salt-tolerant alkaline phosphatase from the eggs of the sea urchin *Strongylocentrotus intermedius*. Appl. Biochem. Microbiol..

[47] Aisa E., Aisa M.C., Ambrosini V., Giovannini E. (1982). Alkaline phosphatase in *Asterias bispinosa*: Partial purification and characterization. Comp. Biochem. Physiol. Part B Comp. Biochem..

[48] Asgeirsson B., Hartemink R., Chlebowski J.F. (1995). Alkaline phosphatase from Atlantic cod (*Gadus morhua*). Kinetic and structural properties which indicate adaptation to low temperatures. Comp. Biochem. Physiol. Part B Biochem. Mol. Biol..

[49] Blasco J., Puppo J., Sarasquete M.C. (1993). Acid and alkaline phosphatase activities in the clam *Ruditapes philippinarum*. J. Mar. Biol..

[50] Gelman A., Mokady S., Cogan U. (1995). The thermal properties of intestinal alkaline phosphatese of three kinds of deep-water fish. Comp. Biochem. Physiol. B Comp. Biochem..

[51] McComb R.B., Bowers G.N., Posen S. (1979). Measurement of Alkaline Phosphatase Activity. Alkaline Phosphatase.

